# Erratum to “Development of a Rapid and Precise Method of Digital Image Analysis to Quantify Canopy Density and Structural Complexity”

**DOI:** 10.1155/2017/3493691

**Published:** 2017-01-18

**Authors:** Anne E. Goodenough, Andrew S. Goodenough

**Affiliations:** ^1^Department of Natural and Social Sciences, Francis Close Hall, University of Gloucestershire, Swindon Road, Cheltenham, Glos GL50 4AZ, UK; ^2^St Briavels, Forest of Dean, Gloucestershire, UK

In the article titled “Development of a Rapid and Precise Method of Digital Image Analysis to Quantify Canopy Density and Structural Complexity,” [1] the description of the Supplementary Materials was incorrect. The correct description should be as follows.

The suite of computer software comprising CanopyDigi is presented here as supplementary material as a zip file. The entire contents of the zip file, including all files and the folder structure, should be extracted to a root directory (e.g., C drive) in the format C:∖CanopyDigi (not C:∖CanopyDigi∖CanopyDigi). The software can run from any root drive, including an external drive.
